# Vitamin D status during pregnancy modulates the effect of pre-pregnancy obesity on gestational diabetes mellitus risk: a birth cohort study

**DOI:** 10.1017/S1368980026101906

**Published:** 2026-01-26

**Authors:** Ali H. Ziyab, Abdullah Al-Taiar, Reem Al-Sabah, Majeda S. Hammoud, Saeed Akhtar

**Affiliations:** 1 Department of Community Medicine and Behavioral Sciences, College of Medicine, Kuwait University, Safat, Kuwait; 2 Joint School of Public Health, Macon & Joan Brock Virginia Health Sciences at Old Dominion University, Norfolk, VA, USA; 3 Department of Pediatrics, College of Medicine, Kuwait University, Safat, Kuwait

**Keywords:** Gestational diabetes mellitus, Obesity, Vitamin D, 25-hydroxyvitamin D, Pregnancy, Maternal age, Interaction, Effect modification

## Abstract

**Objective:**

To determine whether gestational vitamin D status modulates the effect of pre-pregnancy obesity on gestational diabetes mellitus (GDM) risk while stratifying by maternal age.

**Design:**

Birth cohort.

**Setting:**

A major maternity hospital in Kuwait.

**Participants:**

Pregnant women in their second/third trimester of gestation were enrolled. Pre-pregnancy BMI (kg/m^2^) was categorised as under/normal weight (< 25·0), overweight (25·0 to < 30·0) and obesity (≥ 30·0). Gestational 25-hydroxyvitamin D concentrations were categorised as deficiency (< 50 nmol/l) or insufficiency/sufficiency (≥ 50 nmol/l). GDM status was ascertained according to international guidelines. Adjusted OR (aOR) and 95 % CI were estimated using logistic regression.

**Results:**

Data from 957 pregnant women were analysed, with GDM affecting 166 (17·4 %) pregnancies. Pre-pregnancy obesity and gestational vitamin D deficiency were ascertained in 275 (28·7 %) and 533 (55·7 %) pregnant women, respectively. The association between pre-pregnancy obesity and GDM risk differed according to maternal age and gestational vitamin D status (*P*_interaction[BMI × age × vitamin D]_ = 0·041). Among women aged < 35 years (*n* 710), pre-pregnancy obesity compared to under/normal weight was associated with increased GDM risk among women with gestational vitamin D deficiency (aOR: 2·72, 95 % CI: 1·18, 6·23) and vitamin D insufficiency/sufficiency (2·55, 1·15, 5·62). In contrast, among women aged ≥ 35 years (*n* 247), pre-pregnancy obesity compared to under/normal weight was associated with increased GDM risk among women with gestational vitamin D deficiency (6·92, 1·45, 33·04), but not among women with vitamin D insufficiency/sufficiency (1·13, 0·36, 3·56).

**Conclusions:**

Gestational vitamin D status modulates the effect of pre-pregnancy obesity on GDM risk in an age-specific manner.

Gestational diabetes mellitus (GDM), a common pregnancy complication affecting about 14 % of pregnancies globally^(1)^, is described as the onset or first recognition of hyperglycemia (high blood glucose) during pregnancy that resolves almost immediately after delivery^(2)^. Although GDM is a transient condition, it carries short- and long-term consequences for the mother and her child. Common GDM-related immediate pregnancy complications include pre-eclampsia, excessive fetal growth, preterm delivery and caesarean section delivery^(2,3)^. A landmark multinational epidemiological study, ‘Hyperglycemia and Adverse Pregnancy Outcome’, demonstrated that elevated gestational blood glucose levels below standard diabetes diagnostic thresholds are associated with a continuously increasing risk of various maternal and fetal outcomes, including CVD^(4)^. The life course implications for women and children exposed to GDM extend beyond an increased risk of developing diabetes and obesity, with evidence indicating an increased risk of developing CVD^(5,6)^, among others. Two large meta-analyses have reported an increased risk of CVD in women with a prior history of GDM compared to women with no prior history of GDM that is not solely mediated through the development of diabetes or conventional cardiometabolic risk factors^(7,8)^. Moreover, a Mendelian randomisation study further demonstrated a potential causal association between GDM and CVD risk^(9)^. Hence, identifying and mitigating the effects of modifiable risk factors of GDM is a priority for maternal and child health.

Observational studies have identified multiple modifiable and non-modifiable risk factors for GDM development. Non-modifiable risk factors include increased maternal age at pregnancy, family history of diabetes, non-Caucasian race/ethnicity and genetic predisposition^(2,10)^. Whereas modifiable risk factors include pre-pregnancy overweight/obesity, cigarette smoking, physical inactivity and dietary factors^(2,10)^. Among these, pre-pregnancy overweight/obesity, is the most consistently and strongly associated risk factor with GDM development^(11,12)^, affecting almost half of pregnancies globally^(13)^. Emerging evidence from Mendelian randomisation studies further supports the potential causal effect of maternal obesity and body composition on GDM risk^(14,15)^. Although the role of dietary factors and physical activity in the development of GDM is gaining scientific and clinical attention as they can be modified, their effects remain inconclusive^(10,16)^.

Vitamin D (calciferol), a fat-soluble vitamin primarily synthesised in the skin in response to sunlight exposure and which can also be obtained through diet or supplements, has a well-established role in skeletal health, with accumulating evidence suggesting its involvement in many non-skeletal conditions^(17,18)^. Vitamin D has been shown to influence pancreatic *β*-cell function and insulin sensitivity^(19,20)^, which may explain the observed association between vitamin D deficiency and GDM risk, as summarised in large meta-analysis studies^(21,22)^. Existing evidence indicates that increased BMI is associated with decreased 25-hydroxyvitamin D (25(OH)D [calcidiol]; biomarker of vitamin D status) concentrations^(23,24)^. Hence, obesity combined with vitamin D deficiency may synergistically increase the risk of GMD. Given the inconsistent findings from prior studies on the role of vitamin D in mitigating obesity-induced insulin resistance, the metabolic mechanism underlying diabetes/GDM development^(25,26)^, we sought to determine whether vitamin D status during pregnancy modulates the effect of pre-pregnancy overweight/obesity on GDM risk while stratifying by maternal age (a non-modifiable risk factor for GDM) using data from a prospective birth cohort study.

## Methods

### Study design, setting and participants

Pregnant women in their second or third trimester were enrolled in the Kuwait Birth Cohort (KBC) study that aimed to prospectively investigate the influence of gestational vitamin D status and other exposures on maternal and child health outcomes. Study participants were recruited between 2017 and 2021 from a major governmental hospital (Maternity Hospital, Sabah Health Area, Kuwait) that covers about one-third of all deliveries in the State of Kuwait. A total of 1108 pregnant women were enrolled, with the current analysis being restricted to women with available information on pre-pregnancy BMI, GDM status and vitamin D status, and participants with a prior history of type 1/type 2 diabetes were excluded. After applying the aforementioned inclusion and exclusion criteria, data from a total of 957 pregnant women were analysed in this report. More details on the study setting and participants can be found in a previous publication^(27)^. The study was reviewed and approved by the ethics committee at the Ministry of Health, Kuwait (Ref: project 173/2014; date: 14 February 2017) and the Institutional Review Board at Old Dominion University (Ref: 1517949). Written informed consent was obtained from all subjects prior to enrolment in the study.

### Gestational diabetes mellitus ascertainment

GDM status was self-reported by pregnant women at the time of enrolment by asking the following question: ‘During this pregnancy, were you diagnosed with GDM by a physician?’ After delivery, self-reported GDM status was validated by review of medical records by the study clinician. The hospital from which we have enrolled pregnant women uses the International Association of Diabetes and Pregnancy Study Groups (IADPSG) diagnostic criteria to ascertain GDM status^(28)^. The IADPSG recommendation for diagnosing GDM is to use a 75 g oral glucose tolerance test performed at 24–28 weeks of gestation in pregnant women with no prior history of diabetes. A GDM diagnosis was made if fasting plasma glucose was ≥ 5·1 mmol/l (92 mg/dl) and/or 1-h plasma glucose was ≥ 10·0 mmol/l (180 mg/dl) and/or 2-h plasma glucose was ≥ 8·5 mmol/l (153 mg/dl)^(28)^.

### Vitamin D status

Serum concentration of 25(OH)D (biological marker of vitamin D status) was measured in fresh blood samples collected at time of enrolment (second or third trimester of pregnancy) using electrochemiluminescence immunoassay with the Cobas e601 analyser (Roche Diagnostics GmbH), commercial kit (Cat. # 9038078190). Inter-assay CV ranged approximately between 2·8 % and 9·8 %, while intra-assay CVs ranged approximately between 2·3 % and 7·4 %, with better precision at higher concentrations. Given the variability between 25(OH)D assays and to harmonise results to the reference LC–MS/MS scale^(29)^, we retrospectively standardised our 25(OH)D values using a published Passing–Bablok calibration derived on cobas e601 to obtain standardised concentrations^(30)^. Primary analysis used original values; sensitivity analysis using standardised values is shown in online Supplemental Materials. Following the 2011 guidelines of the Endocrine Society, vitamin D status was assessed based on the 25(OH)D serum concentration as severe deficiency < 25·0 nmol/l (< 10 ng/ml), deficiency ≥ 25 to < 50 nmol/l (≥ 10 to < 20 ng/ml), insufficiency ≥ 50 to < 75 nmol/l (≥ 20 to < 30 ng/ml) and sufficiency ≥ 75 nmol/l (≥ 30 ng/ml)^(31)^.

### Pre-pregnancy BMI, maternal age and other study variables

Study questionnaires, collecting data on sociodemographic, lifestyle and dietary factors, and clinical and pregnancy history, were completed through face-to-face interviews with pregnant women, conducted by a trained study interviewer. Pre-pregnancy BMI was calculated by dividing self-reported pre-pregnancy weight in kilograms by measured height in metres squared (kg/m^2^). BMI classifications were defined as underweight (< 18·5 kg/m^2^), normal weight (18·5 to < 25·0 kg/m^2^), overweight (25·0 to < 30·0 kg/m^2^) and obese (≥ 30·0 kg/m^2^). Maternal age at the time of enrolment was categorised as < 35 years or ≥ 35 years (i.e. advanced maternal age) following the current Obstetric Care Consensus published by the American College of Obstetricians and Gynecologists and the Society for Maternal-Fetal Medicine^(32)^. Moreover, study participants reported whether they have smoked tobacco (cigarettes and/or waterpipe) in the past 9 months and reported exposure to household secondhand smoking during this pregnancy. Data on age at first pregnancy, total number of previous pregnancies, ever had an abortion or miscarriage, and current use of supplements and vitamins were collected. Moreover, physical activity during the current pregnancy was measured using the standardised and validated ‘pregnancy physical activity questionnaire’^(33,34)^, which was used to estimate metabolic equivalent of task that approximates total daily energy expenditure.

### Statistical analysis

Statistical analyses were conducted using SAS 9.4 (SAS Institute). Descriptive analyses were conducted to calculate frequencies and proportions of categorical variables, and medians and interquartile ranges (IQR, 25th percentile and 75th percentile) were estimated to describe continuous variables. For all association analyses, a two-sided *P*-value < 0·05 was considered the cut-off point for statistical significance. Univariable associations between categorical variables were evaluated using χ^2^ test to assess differences in frequencies. Moreover, the Wilcoxon rank-sum test (to compare two groups of a categorical variable) and the Kruskal–Wallis test (to compare three or more groups of a categorical variable) were applied to test for associations between an independent categorical variable and a continuous outcome variable.

Multivariable analyses were conducted to evaluate associations between pre-pregnancy BMI (categorical exposure variable) and GDM status (dichotomous outcome variable) by applying binary logistic regression models to estimate adjusted OR (aORs) and their 95 % CIs. The pre-pregnancy BMI variable was modelled as a categorical variable (obesity, overweight and under/normal weight [reference group]). To test whether the association between pre-pregnancy BMI and GDM status is modulated by maternal age (< 35 and ≥ 35 years; effect modifier) and gestational vitamin D status (deficiency [25(OH)D < 50 nmol/l] and insufficiency/sufficiency [25(OH)D ≥ 50 nmol/l]; effect modifier), statistical interaction on a multiplicative scale was tested by including a three-way product term (pre-pregnancy BMI × maternal age × vitamin D status) in the logistic regression models. Subsequently, associations between pre-pregnancy BMI and GDM status were stratified by age group and vitamin D status. Moreover, associations between pre-pregnancy obesity and GDM status were assessed at different concentrations of 25(OH)D (modelled as a continuous variable) while stratifying by age group. Variables that showed a possible association (i.e. *P*-value < 0·25) with GDM status, pre-pregnancy BMI and/or vitamin D status in the univariable analysis were considered as potential confounders. Subsequently, variables that were deemed as potential confounders (as described in the aforementioned sentence) were included in the model selection process. Stepwise backward selection procedure was applied to build a parsimonious logistic regression model that includes a set of potential confounders, with a *P*-value < 0·2 being the criterion for a variable to be retained in the final model. The Hosmer–Lemeshow goodness-of-fit test was used to evaluate model adequacy.

### Sensitivity analysis

We have re-evaluated the regression models and associations using the standardised 25(OH)D values as described in the ‘Vitamin D status’ subsection of the methods section.

## Results

### Description of study sample

A total of 1108 women in their second or third trimester of pregnancy (median weeks of gestation: 35·0, IQR: 30·0–37·0) were enrolled in the KBC study. Table 1 presents the characteristics of the total sample (*n* 1108), the analytical sample (*n* 957) and the excluded sample (*n* 151). Compared to the analytical sample, participants excluded due to missing data had lower educational attainment, were less likely to be employed and had lower exposure to household secondhand smoke (Table 1). Nonetheless, the analytical and excluded samples did not differ significantly with regard to the main study variables: GDM status, 25(OH)D status, pre-pregnancy BMI and age.

**Table 1.** Characteristics and lifestyle factors of the total enrolled sample, analytical sample and excluded sample of pregnant women

	Total sample (*n* 1108)		Analytical sample^*^ (*n* 957)		Excluded sample^†^ (*n* 151)		*P*-value
	*n*	%	*n*	%	*n*	%	
Age (years)							
< 25	117	10·6	101	10·6	16	10·6	0·090^‡^
25 to < 30	330	29·8	298	31·1	32	21·2	
30 to < 35	368	33·2	311	32·5	57	37·7	
≥ 35	293	26·4	247	25·8	46	30·5	
Median IQR	31·2	27·8–35·3	31·0	27·6–35·2	32·2	28·4–35·9	0·051^§^
Gestational age at enrolment (weeks)							
Median IQR	35·0	30·0–37·0	35·0	30·0–37·0	34·0	28·0–36·5	0·160^§^
Missing, *n*	32		21		11		
Nationality							
Kuwaiti	262	23·7	227	23·7	35	23·2	0·884^‡^
Non-Kuwaiti	846	76·3	730	76·3	116	76·8	
Education attainment							
Elementary school or less	29	2·6	18	1·9	11	7·9	<0·001^‡^
High school	249	22·7	216	22·6	33	23·5	
University degree or more	819	74·7	723	75·5	96	68·6	
Missing, *n*	11		–		11		
Employment status							
Employed	492	44·9	443	46·3	49	35·0	0·024^‡^
Unemployed/housewife	581	53·0	492	51·5	89	63·6	
Other	23	2·1	21	2·2	2	1·4	
Missing, *n*	12		1		11		
Smoking in the past 9 months							
Yes	60	5·4	53	5·5	7	4·6	0·649^‡^
No	1048	94·6	904	94·5	144	95·4	
Household secondhand smoking							
Yes	411	37·1	367	38·4	44	29·1	0·030^‡^
No	697	62·9	590	61·6	107	70·9	
Pre-pregnancy BMI (kg/m^2^) category							
Underweight (< 18·5)	24	2·4	23	2·4	1	3·1	0·070^‡^
Normal weight (18·5 to < 25·0)	305	30·8	301	31·5	4	12·5	
Overweight (25·0 to <30·0)	375	37·9	358	37·4	17	53·1	
Obesity (≥ 30·0)	285	28·8	275	28·7	10	31·3	
Median IQR	27·2	23·9–30·5	27·1	23·9–30·5	27·9	26·3–30·7	0·187^§^
Missing, *n*	119		–		119		
Gestational 25(OH)D status (nmol/l)							
Severe deficiency (< 25·0)	238	21·6	197	20·6	41	27·7	0·230^‡^
Deficiency (≥ 25·0 to < 50·0)	380	34·4	336	35·1	44	29·7	
Insufficiency (≥ 50·0 to < 75·0)	227	20·5	199	20·8	28	18·9	
Sufficiency (≥ 75·0)	260	23·5	225	23·5	35	23·7	
Median IQR	44·0	27·6–72·5	44·9	28·1–72·2	39·5	23·8–73·1	0·233^§^
Missing, *n*	3		–		3		
Gestational diabetes mellitus							
Yes	187	17·2	166	17·4	21	16·2	0·735^‡^
No	900	82·8	791	82·6	109	83·8	
Missing, *n*	21		–		21		

IQR, interquartile range (25th, 75th percentiles); 25(OH)D, 25-hydroxyvitamin D.

* This includes participates with available information on pre-pregnancy BMI, gestational diabetes status, and vitamin D status and excludes participants with a prior history of type1/type2 diabetes.

† This includes participants with missing information on pre-pregnancy BMI, gestational diabetes status or vitamin D status and includes participants with a prior history of type1/type2 diabetes. This sample includes participants that were excluded from the analytical sample.

‡ Calculated using χ^2^ test to compare distribution of variables across the analytical sample and the excluded sample. If any cell expected count was less than five observations, Fisher’s exact test was used.

§ Calculated using the Wilcoxon rank-sum test to compare median of variables across the analytical sample and the excluded sample.

In the analytical sample, the median age of study subjects at the time of enrolment was 31·0 (IQR: 27·6–35·2) years, with 25·8 % aged ≥ 35 years. Cigarette and/or waterpipe smoking during pregnancy was reported by 5·5 % of the participants, whereas 38·4 % of the participants reported current exposure to household secondhand smoke. Pre-pregnancy overweight and obesity were ascertained in 37·4 % and 28·7 % of the enrolled women, respectively. The prevalence of GDM was estimated to be 17·4 % (95 % CI: 15·0, 19·7) in the analytical study sample (Table 1).

According to 25(OH)D concentrations measured during pregnancy, 20·6 % of the participants had severe vitamin D deficiency and 35·1 % had vitamin D deficiency. While 20·8 % and 23·5 % of the enrolled pregnant women had insufficient and sufficient vitamin D based on 25(OH) concentrations, respectively (Table 1). After standardising 25(OH)D concentrations as described in the methods section, the prevalence of severe deficiency dropped to 10·9 %, while 37·0 %, 23·4 % and 28·7 % of participants had deficient, insufficient and sufficient vitamin D according to the standardised 25(OH)D concentrations, respectively (data not shown).

### Univariable associations

Table 2 shows the prevalence of GDM and concentrations of 25(OH)D and pre-pregnancy BMI according to personal characteristics and lifestyle factors. Increased maternal age was associated with increased GDM prevalence (age ≥ 35: 26·3 % *v*. age < 25: 8·9 %, *P* < 0·001) and higher concentrations of 25(OH)D and pre-pregnancy BMI. The prevalence of GDM was higher among women with pre-pregnancy obesity compared to those with under/normal weight according to BMI (27·6 % *v*. 10·2 %, *P* < 0·001). Concentrations of 25(OH)D did not vary across pre-pregnancy BMI categories (*P* = 0·307). Moreover, GDM prevalence and pre-pregnancy BMI levels were not associated with vitamin D status (*P* > 0·05; Table 2).

**Table 2.** Prevalence of GDM and levels of gestational 25(OH)D and pre-pregnancy BMI according to personal characteristics and lifestyle factors: univariable analysis

	GDM prevalence		25(OH)D, nmol/l		BMI	
	%	*n*/total	Median	IQR	Median	IQR
Total	17·4	166/957	44·9	28·1–72·2	27·1	23·9–30·5
Age (years)						
< 25	8·9	9/101	34·9	22·0–64·5	23·9	21·9–28·4
25 to < 30	15·4	46/298	42·6	26·1–64·9	26·2	23·0–29·9
30 to < 35	14·8	46/311	49·0	30·6–78·3	27·3	24·5–30·1
≥ 35	26·3	65/247	49·2	29·2–78·0	28·9	25·2–32·1
*P*-value	< 0·001^*^		< 0·001^†^		< 0·001^†^	
Nationality						
Kuwaiti	20·7	47/227	50·1	30·3–77·6	26·8	23·7–30·8
Non-Kuwaiti	16·3	119/730	43·8	27·4–70·7	27·2	23·9–30·5
*P*-value	0·126^*^		0·171^†^		0·391^†^	
Education attainment						
Elementary school or less	22·2	4/18	46·7	35·7–67·7	30·8	25·7–33·5
High school	17·1	37/216	38·2	23·1–59·5	27·1	23·7–30·8
University degree or more	17·3	125/723	47·6	29·9–77·2	27·0	23·9–30·4
*P*-value	0·801^*^		< 0·001^†^		0·157^†^	
Employment status^‡^						
Employed	20·5	91/443	50·0	31·0–79·0	27·2	23·9–30·8
Unemployed/housewife	14·6	75/513	40·9	25·4–65·1	26·9	23·8–30·5
*P*-value	0·016^*^		< 0·001^†^		0·693^†^	
Smoking in the past 9 months						
Yes	20·8	11/53	49·2	30·2–78·2	26·3	24·0–30·5
No	17·2	155/904	44·4	27·9–72·1	27·2	23·9–30·5
*P*-value	0·501^*^		0·439^†^		0·553^†^	
Household secondhand smoking						
Yes	15·8	58/367	42·5	26·1–66·9	27·1	23·7–30·4
No	18·3	108/590	47·6	29·4–75·9	27·1	24·0–30·8
*P*-value	0·320^*^		0·026^†^		0·244^†^	
Age of menarche (years)						
8 to < 12	21·5	28/130	44·4	26·3–72·5	28·3	24·9–31·2
12 to < 13	15·7	42/268	45·1	29·3–70·5	27·3	23·9–30·3
13 to < 14	18·9	52/275	45·2	28·7–76·1	26·9	24·2–30·8
≥ 14	15·2	41/270	43·2	25·1–70·5	26·0	23·2–30·1
*P*-value	0·324^*^		0·661^†^		0·019^†^	
Pre-pregnancy BMI (kg/m^2^) category^§^						
Under/normal weight (< 25·0)	10·2	33/324	44·3	27·5–75·8	–	
Overweight (25·0 to < 30·0)	15·9	57/358	46·6	30·0–75·4	–	
Obesity (≥ 30·0)	27·6	76/275	43·3	26·9–66·5	–	
*P*-value	< 0·001^*^		0·307^†^		–	
Vitamin D status (nmol/l)						
Deficiency (< 50·0)	16·3	87/533	–		26·7	23·9–30·3
In-/sufficiency (≥ 50·0)	18·6	79/424	–		27·3	23·8–30·8
*P*-value	0·349^*^		–		0·486^†^	
Season of blood draw						
Summer	15·0	24/160	40·4	20·3–60·6	27·5	23·4–31·1
Fall	19·0	63/331	43·3	26·3–73·6	27·3	24·2–31·2
Winter	13·5	32/237	48·6	30·9–79·2	27·2	23·9–30·5
Spring	20·6	47/228	48·2	29·7–78·9	26·1	23·5–29·8
*P*-value	0·144^*^		< 0·001^†^		0·083^†^	

GDM, gestational diabetes mellitus; 25(OH)D, 25-hydroxyvitamin D; IQR, interquartile range (25th, 75th percentiles).

* Calculated using χ^2^ test. If any cell expected count was less than five observations, Fisher’s exact test was used.

† Calculated using the Wilcoxon rank-sum test when comparing the medians of two groups and the Kruskal–Wallis test when comparing medians of three or more groups.

‡ The ‘other’ category of the employment status (*n* 21) was merged with the ‘unemployed/wife’ category due to small sample size.

§ The ‘underweight’ (*n* 23) category of the BMI status was merged with the ‘normal weight’ category due to small cell size.

Table 3 shows the prevalence of GDM and concentrations of 25(OH)D and pre-pregnancy BMI according to history of prior pregnancies, physical activity status, fasting and use of dietary supplements during the current pregnancy, and personal and familial clinical history. Increased age at first pregnancy was associated with increased GDM prevalence and higher concentrations of 25(OH)D. Higher number of prior pregnancies was associated with increased GMD prevalence and elevated pre-pregnancy BMI, while concentrations of 25(OH)D showed a decreasing trend as the number of prior pregnancies increased. Current use of dietary supplements and vitamins was associated with increased concentrations of 25(OH)D (45·5 *v*. 30·8 nmol/l, *P* < 0·001). Moreover, parental/sibling history of diabetes and maternal/sister history of GDM were associated with increased prevalence of GDM (Table 3).

**Table 3.** Prevalence of GDM and levels of gestational 25(OH)D and pre-pregnancy BMI according to history of prior pregnancies, physical activity level, fasting history and use of supplements and vitamins during the current pregnancy, and personal and familial clinical history: univariable analysis

	GDM prevalence		25(OH)D, nmol/l		BMI	
	%	*n*/total	Median	IQR	Median	IQR
Age at first pregnancy (years)						
Tertile 1: < 23 years	17·6	48/273	39·4	24·7–63·6	27·4	23·4–31·6
Tertile 2: ≥ 23 to < 27	14·1	51/362	45·1	28·9–72·7	26·9	24·0–29·8
Tertile 3: ≥ 27	21·0	66/315	54·0	31·0–79·5	27·1	23·9–30·8
*P*-value	0·063^*^		< 0·001^†^		0·510^†^	
Total number of previous pregnancies						
Zero	9·4	18/192	46·9	26·0–80·0	24·5	22·0–27·5
1–3	17·6	99/564	45·8	29·5–70·4	27·3	24·1–30·5
≥ 4	24·2	48/198	40·1	23·6–70·4	28·9	25·7–32·8
* P*-value	< 0·001^*^		0·079^†^		< 0·001^†^	
Treatment to assist with pregnancy						
Yes	28·0	26/93	60·5	35·2–77·5	27·3	24·7–32·4
No	16·2	140/862	43·5	27·1–71·4	27·1	23·8–30·4
*P*-value	0·005^*^		0·003^†^		0·052^†^	
Ever had an abortion or miscarriage						
Yes	21·5	78/363	43·9	29·2–69·7	27·8	24·8–31·3
No	14·8	88/594	45·1	27·1–75·9	26·7	23·2–30·1
*P*-value	0·008^*^		0·455^†^		< 0·001^†^	
Physical activity (total MET)						
Low	20·6	65/316	48·5	30·6–74·8	26·8	24·0–30·9
Moderate	14·6	47/322	42·9	27·4–71·4	27·2	23·7–30·5
High	16·4	51/311	43·3	26·2–69·9	27·2	23·7–30·5
*P*-value	0·123^*^		0·140^†^		0·827^†^	
Fasted during this pregnancy						
Yes	15·6	80/513	41·4	25·4–69·0	26·9	23·7–30·5
No	19·3	85/441	49·2	30·6–77·2	27·1	24·2–30·5
*P*-value	0·134^*^		< 0·001^†^		0·339^†^	
Current use of supplements and vitamins						
Yes	17·7	160/902	45·5	28·8–72·7	27·1	23·9–30·5
No	10·0	5/50	30·8	19·3–55·0	27·0	23·8–30·1
*P*-value	0·183^*^		< 0·001^†^		0·806^†^	
Diagnosed hypertension						
Yes	28·1	18/64	44·2	29·8–69·4	31·7	27·7–34·9
No	16·6	148/893	45·0	28·1–72·7	26·8	23·7–30·2
*P*-value	0·018^*^		0·922^†^		< 0·001^†^	
Parental/sibling history of diabetes						
Yes	22·7	95/419	48·4	30·5–78·7	27·6	24·5–31·6
No	13·3	71/535	41·9	26·3–68·2	26·7	23·4–30·0
*P*-value	< 0·001^*^		0·007^†^		< 0·001^†^	
Maternal/sisters’ history of GDM						
Yes	30·8	12/39	50·1	25·3–72·1	26·7	24·5–29·4
No	16·8	154/915	44·3	28·1–72·5	27·1	23·8–30·5
*P*-value	0·025^*^		0·735^†^		0·945^†^	

GDM, gestational diabetes mellitus; 25(OH)D, 25-hydroxyvitamin D; IQR, interquartile range (25th, 75th percentiles); MET, metabolic equivalent of task.

* Calculated using χ^2^ test. If any cell expected count was less than five observations, Fisher’s exact test was used.

† Calculated using the Wilcoxon rank-sum test when comparing the medians of two groups and the Kruskal–Wallis test when comparing medians of three or more groups.

### Associations between pre-pregnancy BMI and gestational diabetes mellitus status according to vitamin D status and age group

Results of univariable analysis show that increased pre-pregnancy BMI and increased maternal age were strongly associated with increased GDM prevalence. However, univariable analysis indicated no association between gestational vitamin D status and GDM prevalence (Table 2). To assess whether vitamin D status (insufficiency/sufficiency or deficiency) and maternal age (< 35 or ≥ 35 years) modulate the effect of pre-pregnancy overweight/obesity on GDM risk, a three-way statistical interaction term was tested (pre-pregnancy BMI × maternal age × vitamin D status). The three-way interaction effect was statistically significant in the multivariable logistic regression model (*P*_interaction_ = 0·041) that adjusted for the effects of employment status, receiving treatment to assist with pregnancy, current use of supplements and vitamins, parental/sibling history of diabetes, maternal/sister history of GDM, total number of previous pregnancies and physical activity (total metabolic equivalent of task) during pregnancy (Table 4). Subsequently, adjusted associations between pre-pregnancy BMI categories and GDM status were evaluated according to gestational vitamin D status and maternal age groups (Table 4). Among pregnant women aged < 35 years, pre-pregnancy overweight was not associated with increased GDM risk, neither among those with deficient vitamin D status (aOR: 1·98, 95 % CI: 0·88, 4·45) nor those with insufficient/sufficient vitamin D status (aOR: 0·74, 95 % CI: 0·32, 1·72). Among pregnant women aged < 35 years, the effect of pre-pregnancy obesity on GDM risk was not modulated by vitamin D status, with pre-pregnancy obesity being associated with increased GDM risk among women with deficient (aOR: 2·72, 95 % CI: 1·18, 6·23) and insufficient/sufficient (aOR: 2·55, 95 % CI: 1·15, 5·62) vitamin D status (Table 4). Moreover, among pregnant women aged ≥ 35 years, the effect size of the association between pre-pregnancy overweight and GDM risk was higher among women with deficient vitamin D status (aOR: 3·83, 95 % CI: 0·74, 19·74) than those with insufficient/sufficient vitamin D status (aOR: 1·59, 95 % CI: 0·54, 4·73), although both associations did not gain statistical significance. In contrast, pre-pregnancy obesity was differentially associated with GDM risk among pregnant women aged ≥ 35 years, with obesity being significantly associated with increased GDM risk among women with deficient vitamin D status (aOR: 6·92, 95 % CI: 1·45, 33·04), but not among women with insufficient/sufficient vitamin D status (aOR: 1·13, 95 % CI: 0·36, 3·56; Table 4). In a further analysis assessing the joint effects, we showed that ‘pregnant women aged ≥ 35 years with pre-pregnancy obesity and have deficient vitamin D status’ compared to ‘pregnant women aged < 35 years with normal pre-pregnancy BMI and have insufficient/sufficient vitamin D status’ had an increased risk of developing GDM (aOR: 4·87, 95 % CI: 1·77, 13·42, *P* = 0·002; data not shown).

**Table 4.** Associations between pre-pregnancy BMI categories (exposure variable) and GDM (outcome variable) according to age group (effect modifier) and vitamin D status (effect modifier)

		Vitamin D status (effect modifier)									
		Deficiency (25(OH)D < 50 nmol/l)					Insufficiency/sufficiency (25(OH)D ≥ 50 nmol/l)				
Age category (effect modifier)	BMI category (exposure)	GDM		GDM (outcome)		*P*-value^†^	GDM		GDM (outcome)		*P*-value^†^
		%	*n*/total	aOR^*^	95 % CI		%	*n*/total	aOR^*^	95 % CI	
< 35 years	Normal weight	6·4	10/156	1·00 (Reference)		0·032	12·4	14/113	1·00 (Reference)		0·010
	Overweight	14·1	21/149	1·98	0·88, 4·45		10·9	14/129	0·74	0·32, 1·72	
	Obesity	18·5	20/108	2·72	1·18, 6·23		32·4	23/71	2·55	1·15, 5·62	
≥ 35 years	Normal weight	8·0	2/25	1·00 (Reference)		0·018	23·5	8/34	1·00 (Reference)		0·623
	Overweight	23·3	10/43	3·83	0·74, 19·74		26·0	13/50	1·59	0·54, 4·73	
	Obesity	40·0	25/62	6·92	1·45, 33·04		23·8	10/42	1·13	0·36, 3·56	

GDM, gestational diabetes mellitus; 25(OH)D, 25-hydroxyvitamin D; aOR, adjusted OR.

In the multivariable logistic regression model, *P*-value for the three-way interaction term (pre-pregnancy BMI × maternal age × vitamin D status) = 0·041.

* Adjusted for employment status, receiving treatment to assist with pregnancy, current use of supplements and vitamins, parental/sibling history of diabetes, maternal/sisters’ history of GDM, total number of previous pregnancies and physical activity (total metabolic equivalent of task) during pregnancy.

† *P*-value obtained from the global Wald test assessing whether all statistical parameters are equal to zero.

**Figure 1.**
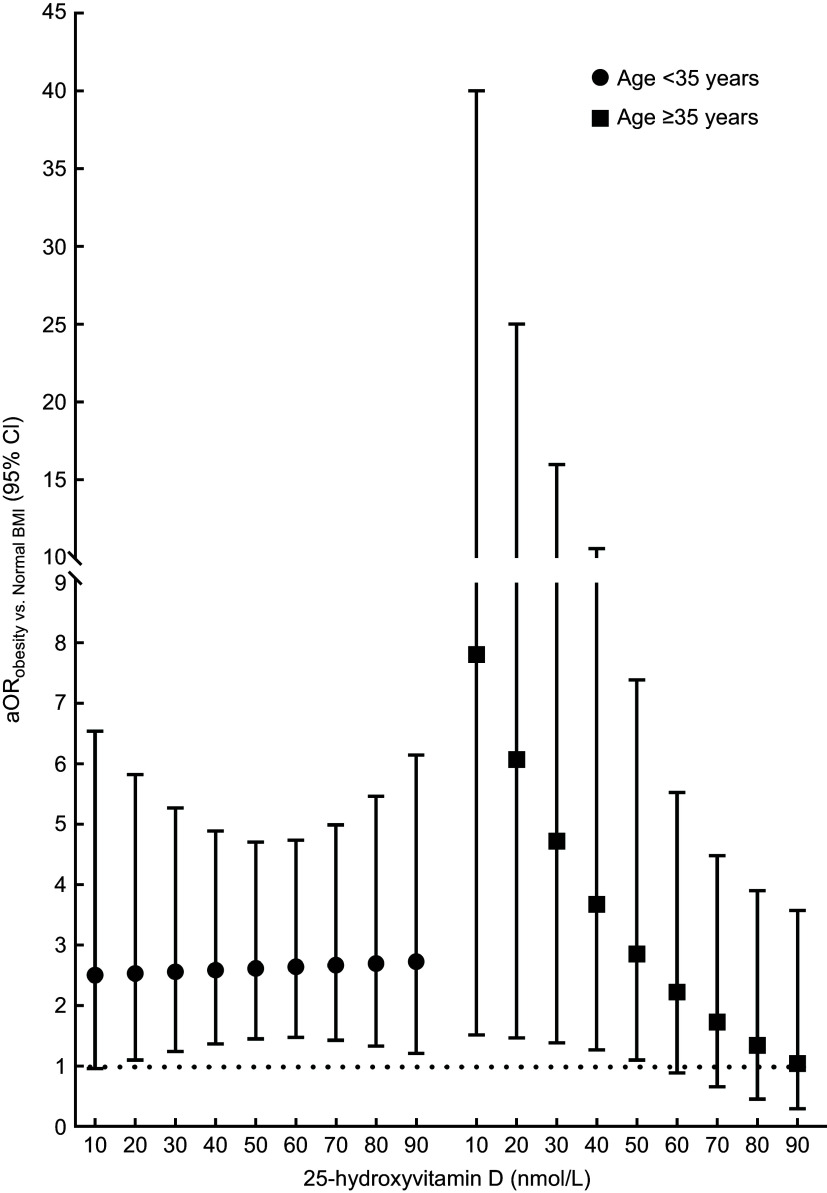
Associations between pre-pregnancy obesity and gestational diabetes mellitus (GDM) according to 25-hydroxyvitmain D concentrations (nmol/l) stratified by maternal age. Adjusted OR (aOR) relating pre-pregnancy obesity with GDM (y-axis) at different concentrations of 25-hydroxyvitmain D (nmol/l) stratified by maternal age (< 35 *v*. ≥ 35 years). The aOR along with their 95 % CI were estimated while comparing the odds of GDM in pregnant women with pre-pregnancy obesity (BMI ≥ 30·0 kg/m^2^) to the odds of GDM among pregnant women with normal pre-pregnancy BMI (< 25·0 kg/m^2^). The estimated aOR were adjusted for employment status, receiving treatment to assist with pregnancy, current use of supplements and vitamins, parental/sibling history of diabetes, maternal/sisters’ history of GDM, total number of previous pregnancies and physical activity (total metabolic equivalent of task) during pregnancy. The dotted horizontal line refers to the null value of ‘1’.

In additional analyses, we assessed associations between pre-pregnancy obesity and GDM status at different concentrations of 25(OH)D (modelled as a continuous variable) while stratifying by age group (Figure 1). Results of these analyses showed that the association between pre-pregnancy obesity and GDM status did not differ as 25(OH)D concentrations changed among pregnant women aged < 35 years. Whereas, among pregnant women aged ≥ 35 years, the magnitude of the association between pre-pregnancy obesity and GDM status showed a decreasing pattern as 25(OH)D concentrations increased (Figure 1). For instance, at 10 nmol/l of 25(OH)D, pre-pregnancy obesity compared to normal BMI was associated with increased GDM risk (aOR: 7·81, 95 % CI: 1·52, 40·06, *P* = 0·014) among pregnant women aged ≥ 35 years, with this effect nearly reaching the null at 90 nmol/l of 25(OH)D (aOR: 1·05, 95 % CI: 0·31, 3·59, *P* = 0·934; Figure 1). These findings further corroborate the reported effect modification in Table 4 by vitamin D status and age group.

Results of sensitivity analysis that used the standardised 25(OH)D concentrations to replicate the main analysis presented in Table 4 are shown in the online supplementary material, Supplemental Table S1. Similar to the results found in Table 4, the three-way interaction effect was statistically significant in the multivariable logistic regression model (*P*_interaction_ = 0·017). Moreover, similar magnitudes and directions of associations were identified (see online supplementary material, Supplemental Table S1).

## Discussion

Our study demonstrated that the association between pre-pregnancy obesity and GDM risk varies according to maternal age and gestational vitamin D status. Specifically, after controlling for the effect of potential confounders, among older pregnant women (aged ≥ 35 years) with vitamin D deficiency, obesity was strongly associated with increased risk of GDM, whereas obesity was not associated with GDM risk among older pregnant women with sufficient or insufficient vitamin D status. Among younger pregnant women (aged < 35 years), obesity was associated with increased GDM risk irrespective of vitamin D status. These findings indicate that the effect of pre-pregnancy obesity on GDM risk might be mitigated in older pregnant women by increasing 25(OH)D concentrations.

Pre-pregnancy overweight/obesity complicates a large proportion of pregnancies globally^(13)^ and is associated with a wide range of pregnancy, birth and postpartum adverse health outcomes, including increased risk of GDM^(35)^. Women entering pregnancy with overweight/obesity are more likely to develop GDM than women with normal pre-pregnancy weight, most probably due to the asymptomatic pre-conception obesity-induced metabolic dysfunction that is characterised by pancreatic *β*-cells defects and increased insulin resistance^(2)^. On the other hand, vitamin D deficiency is common in pregnancy^(36)^, which may predispose affected pregnant women to a higher risk of GDM through its role in regulating pancreatic *β*-cells function and insulin responsiveness to glucose^(37)^. Large meta-analyses of observational studies have shown that vitamin D deficiency increases the risk of GDM^(21,22)^. However, contradictory findings have been reported regarding the effectiveness of vitamin D supplementation on GDM risk, gestational glycaemic control and insulin sensitivity. A multicentre randomised controlled trial showed that vitamin D supplementation was not effective in reducing the risk of GDM among overweight/obese pregnant women^(38)^. In contrast, a meta-analysis of seven randomised controlled trials reported that high dose of vitamin D supplementation (> 2000 IU/d) compared to lower dose of supplementation was associated with reduced risk of GDM^(39)^. Moreover, a meta-analysis of nineteen controlled clinical studies has reported that vitamin D supplementation among pregnant women with established GDM diagnosis was associated with improved glycaemic control and reduced adverse maternal–neonatal outcomes^(40)^. A randomised controlled trial among obese subjects showed that vitamin D supplementation was associated with improved insulin sensitivity^(25)^. Collectively, increased concentrations of 25(OH)D may lower the risk of GDM. Nonetheless, the reported inconsistent findings in previous studies might be due to the use of different methods of diagnosing GDM and measuring 25(OH)D, confounding in observational studies, different characteristics of study populations (e.g., overweight/obese, vitamin D deficient and older/younger participants) and/or not assessing effect modification (can mask the identification of associations in subgroups).

In the current study, we assessed the association of pre-pregnancy overweight/obesity with GDM risk while considering effect modification by maternal age and vitamin D status. Our results indicate that both maternal age and vitamin D status modified the association between pre-pregnancy overweight/obesity and GDM. Specifically, among pregnant women aged ≥ 35 years, the effect of pre-pregnancy obesity on GDM risk was highly pronounced among pregnant women with vitamin D deficiency (25(OH)D < 50 nmol/l), while the effect of pre-pregnancy obesity on GDM risk was attenuated among pregnant women aged ≥ 35 years with vitamin D insufficiency/sufficiency (25(OH)D ≥ 50 nmol/l). Among pregnant women aged < 35 years, a change in vitamin D status from deficient to insufficient/sufficient attenuated the effect of overweight on GDM risk but did not attenuate the effect of obesity on GDM risk. Our findings indicate that in older pregnant women, increasing 25(OH)D concentrations may mitigate the adverse effect of pre-pregnancy obesity on GDM risk. While among younger pregnant women, correcting vitamin D deficiency may alleviate the effect of overweight on GDM risk. Biologically, overweight/obesity is associated with increased insulin resistance; hence, women with pre-pregnancy overweight/obesity enter pregnancy with a high-risk state for developing GDM. Vitamin D has the potential to counter the adverse metabolic effects of obesity by reducing obesity-induced inflammation and enhancing insulin sensitivity (glucose uptake) through stimulating insulin receptor expression and activation of glucose transporters^(37,41)^.

Few prior studies have assessed the effect modification of pre-pregnancy overweight/obesity on the association between vitamin D and the risk of GDM with inconsistent findings. Arnold et al. showed that the association between vitamin D insufficiency and GDM risk was stronger among women with pre-pregnancy overweight/obesity than among women with normal BMI; nonetheless, the interaction term was not statistically significant^(42)^. Similarly, Li et al. reported an inverse association between vitamin D status and GDM risk in a birth cohort of twin pregnancies, with the association being more pronounced among overweight women^(43)^. Shao et al. identified a statistically significant interaction between pre-pregnancy BMI and vitamin D measured in the second trimester of pregnancy on GDM risk, with vitamin D deficiency in the second trimester being associated with GDM risk among women with pre-pregnancy overweight/obesity, but not among women with underweight/normal pre-pregnancy BMI^(44)^. Moreover, Ou et al. showed that the association between vitamin D status and insulin sensitivity index is stronger among subjects with overweight/obesity compared with those with underweight/normal BMI^(45)^. On the contrary, Xia et al. reported that the association between vitamin D deficiency and GDM risk was not modified by pre-pregnancy BMI, maternal age, race/ethnicity, physical activity, parity or family history of diabetes^(46)^. In terms of effect modification by maternal age, a subgroup analysis in a meta-analysis study showed that high compared to low 25(OH)D concentrations were associated with reduced risk of GDM only among women aged > 30 years^(22)^. The aforementioned studies support our findings by showing that pre-pregnancy overweight/obesity coupled with vitamin D deficiency synergistically increased the risk of GDM. We only identified a single study that supported our observation of effect modification by maternal age, which showed that older pregnant women may benefit more from increased 25(OH)D concentrations with regard to GDM risk^(22)^. To our knowledge, our study is the first to identify a three-way interaction between pre-pregnancy BMI, maternal age, and gestational vitamin D status on GDM risk. Hence, future studies are needed to corroborate our findings and investigate the complex interplay between modifiable and non-modifiable risk factors on the risk of GDM.

Strengths of the current study include the large sample size, prospective assessments and data on a wide range of potential exposures and confounders. Nonetheless, our findings should be interpreted in light of the following limitations. Given that pregnant women were enrolled during their second or third trimester of pregnancy (i.e. past the diagnostic period for GDM, 24–28 weeks of gestation), GDM status in the current study was based on self-reports by the pregnant women. Cross-validation of self-reported GDM status and the medical records showed high agreement (simple kappa coefficient: 0·69; maximum kappa: 0·97; data not shown). Yet, such self-reporting may lead to misclassification of GDM status. Nonetheless, all participants were enrolled from the same centre; hence, they were screened for GDM according to the same diagnostic guidelines and criteria. Therefore, any misclassification will most probably be non-differential and will bias the measures of effect towards the null. Moreover, two prior studies in Kuwait have estimated the prevalence of GDM to be 12·6 % (95 % CI: 10·4, 14·8)^(47)^ and 14·1 % (95 % CI: 11·6, 17·0)^(48)^. These prevalence estimates are close to our estimated prevalence of GDM of 17·4 %. Hence, further confirming the validity of our GDM status assessment. Another limitation of the current analysis is that vitamin D was assessed after the development of GDM. However, we did not identify an association between GDM status and vitamin D status; hence, any suspicion that GDM diagnosis might have influenced vitamin D status is less likely. Therefore, the findings of this report should be interpreted in light of this limitation, and future studies assessing vitamin D status prior to the development of GDM are needed to corroborate our findings. A further limitation is that pre-pregnancy weight was self-reported; nonetheless, prior studies have shown elevated agreement between self-reported pre-pregnancy weight and measured weight at the first prenatal visit^(49,50)^. Moreover, given that some of the analysed subgroups had limited sample sizes (e.g., among the twenty-five women aged ≥ 35 years with deficient vitamin D status, only two had GDM; Table 4), caution should be practised when interpreting the effect measures due to the wide CI. The exclusion of approximately 13·6 % (*n* 151) of the total enrolled sample due to missing data is a further limitation. Our analysis comparing the analytical and excluded samples revealed that excluded participants had lower educational attainment and were less likely to be employed. However, no significant differences were observed regarding the primary study variables, including GDM status, 25(OH)D status, age and pre-pregnancy BMI. This suggests that while the analytical sample may be slightly skewed socio-economically, the exclusion of these participants is unlikely to have biased the associations under investigation.

In conclusion, our study showed that gestational vitamin D status mitigates the effect of pre-pregnancy obesity on GDM risk, with this modulation differing by maternal age. In particular, among pregnant women aged ≥ 35 years, pre-pregnancy obesity was strongly associated with increased risk of GDM among vitamin D-deficient women, with this effect nearly disappearing among women with vitamin D insufficiency/sufficiency. In contrast, among pregnant women aged < 35 years, the effect of pre-pregnancy obesity on GDM risk was not attenuated by vitamin D status. Our findings are of public health importance, suggesting that vitamin D supplementation among older pregnant women may alleviate the effect of pre-pregnancy obesity on GDM risk. Therefore, these findings may pave the way towards developing vitamin D-related risk-stratified GDM intervention and prevention strategies that ultimately aim to improve maternal and child health.

## Supporting information

Ziyab et al. supplementary material Ziyab et al. supplementary material

## Supplementary material

For supplementary material accompanying this paper visit https://doi.org/10.1017/S1368980026101906
