# Supplementary material for: Vitamin D status during pregnancy modulates the effect of pre-pregnancy obesity on gestational diabetes mellitus risk: a birth cohort study
Source: Public Health Nutr. 2026 Jan 26;29(1):e30. doi: 10.1017/S1368980026101906 (PMC12917410; doi:10.1017/S1368980026101906)
Supplement: Ziyab et al. supplementary material [file S1368980026101906sup001.docx]

**Table S1.** Associations between pre-pregnancy body mass index categories (exposure variable) and gestational diabetes mellitus (outcome variable) according to age group (effect modifier) and vitamin D status (effect modifier)

|  |  | **Vitamin D status [effect modifier]** | | | | | | |
| --- | --- | --- | --- | --- | --- | --- | --- | --- |
|  |  | **Deficiency**  **(Standardized 25(OH)D^§^ <50 nmol/L)** | | |  | **Insufficiency/sufficiency**  **(Standardized 25(OH)D^§^ ≥50 nmol/L)** | | |
| **Age category [effect modifier]** | **BMI category [exposure]** | **GDM, % (n/total)** | **GDM [outcome],**  **aOR^*^ (95% CI)** | ***P*-value^†^** |  | **GDM,**  **% (n/total)** | **GDM [outcome],**  **aOR^*^ (95% CI)** | ***P*-value^†^** |
| **<35 years** | Normal weight | 5.9 (8/136) | 1.00 (Reference) | 0.081 |  | 12.0 (16/133) | 1.00 (Reference) | 0.005 |
|  | Overweight | 13.3 (18/135) | 2.03 (0.83, 4.94) |  |  | 11.9 (17/143) | 0.85 (0.39, 1.84) |  |
|  | Obesity | 17.2 (16/93) | 2.52 (1.01, 6.34) |  |  | 31.4 (27/86) | 2.72 (1.31, 5.64) |  |
| **≥35 years** | Normal weight | 5.3 (1/19) | 1.00 (Reference) | 0.017 |  | 22.5 (9/40) | 1.00 (Reference) | 0.353 |
|  | Overweight | 22.9 (8/35) | 5.23 (0.58, 46.97) |  |  | 25.9 (15/58) | 1.74 (0.63, 4.77) |  |
|  | Obesity | 45.8 (22/48) | 13.43 (1.60, 113.0) |  |  | 23.2 (13/56) | 1.15 (0.41, 3.23) |  |

GDM, gestational diabetes mellitus; 25(OH)D, 25-hydroxyvitamine D; BMI, body mass index; aOR, adjusted odds ratio; CI, confidence interval.

^*^ Adjusted for employment status, receiving treatment to assist with pregnancy, current use of supplements and vitamins, parental/sibling history of diabetes, maternal/sisters’ history of GDM, total number of previous pregnancies, and physical activity (total metabolic equivalent of task) during pregnancy.

**^†^** P-value obtained from the global Wald test assessing whether all statistical parameters are equal to zero.

Note: In the multivariable logistic regression model, p-value for the three-way interaction term (pre-pregnancy BMI × maternal age × vitamin D status) = 0.017.

^§^ We have retrospectively standardized the measured 25(OH)D concentrations to the reference LC–MS/MS scale as described in the methods section.
